# Glioblastoma Following Treated Medulloblastoma After 29 Years in the Posterior Fossa: Case Report and Review of Literature

**DOI:** 10.3389/fonc.2022.760011

**Published:** 2022-04-13

**Authors:** Tarek Mesbahi, Hind Zaine, Ismaël Mahazou Abdou, Tarik Chekrine, Souha Sahraoui, Mehdi Karkouri, Abdelhakim Lakhdar

**Affiliations:** ^1^Neurosurgery Department of the IBN ROCHD University Hospital Center, Casablanca, Morocco; ^2^Department of Radiotherapy and Oncology of the IBN ROCHD University Hospital Center, Casablanca, Morocco; ^3^Anatomic Pathology Department of the IBN ROCHD University Hospital Center, Casablanca, Morocco

**Keywords:** glioblastoma, medulloblastoma, meningioma, high-grade glioma, cerebellar neoplasm, long-term effect of radiotherapy

## Abstract

Glioblastoma multiforme (GBM) is a high-grade glioma that may be a rare complication of radiotherapy. We report a case of a patient who was treated for medulloblastoma (MB) of the posterior fossa at the age of 27 years. Twenty-nine years later, at the age of 56 years, he presented with a double-location tumor: supratentorial and in the posterior fossa. Imaging features of the supratentorial location were very suggestive of a meningioma. We operated on the posterior fossa location, which revealed a glioblastoma. Histologically, the tumor cells exhibited characteristics of both GBM and rhabdoid tumor cells. Literature reports of cases of GBM following MB at the same place are very rare, and presenting rhabdoid characteristics is even rarer. This is the first case of MB and GBM at ages 27 and 56 years, respectively. The double-location supratentorial probable meningioma and GBM of the posterior fossa 32 years after MB is the only case reported in the literature. What to do in this case remains a topic of debate, and there are no clear recommendations in the literature.

## Background

The occurrence of a secondary tumor, which is a very rare complication, has increased with the improvement of the survival of patients with brain tumors due to the advances in radiotherapy or intensive chemotherapy (1).

Radiation-induced high-grade glioma may arise following several other tumors, such as glioblastoma multiforme (GBM), medulloblastoma (MB), germinomas, Burkitt’s lymphoma, and treated acute lymphocytic leukemia (2–4). Glioblastoma following MB treated with radiotherapy is an even rarer complication. Schmidbauer et al. (5) reported the first case in 1987 (6). We discovered five more cases of GBM following treated MB in the same location reported (5, 7–10) and two supratentorial (11). These occurred after a long latency period. Previous studies on secondary glioblastoma reported a more aggressive course and poorer prognosis compared with typical GBM (12).

Rhabdoid glioblastoma (RGBM) is rare, but is the most malignant among astrocytic tumors. Accumulating evidence indicates its highly aggressive nature and distinct histopathological features (13).

Radiation-induced meningioma is the most common brain neoplasm known to be caused by ionizing radiation (14, 15). Typically, it is a benign tumor and appears after a long latency period, with an average of 18.1 years. This explains its frequency in the adult population (16).

Here, we report a case of double-location GBM with rhabdoid characteristics following treated MB 29 years later in the posterior fossa and the supratentorial location evoking a probable meningioma.

## Observation

Our patient is a 56-year-old Moroccan man living in Casablanca, married, father of two children, a taxi driver on sick leave for a year, with low socioeconomic status, and is right-handed.

The medical history of the patient was marked by a vitiligo evolving since the age of 13 years, diabetes managed with insulin therapy, a high blood pressure with poor adherence to therapy, and an untreated dyslipidemia. His personal habits were principally alcohol and tobacco addiction. He had no particular family history. He does not present a genetic disease, as well as the members of his family.

He was operated in Lyon, France, in 1992 at the age of 27 for a tumor of the posterior fossa. It was revealed by cerebellar syndrome and intracranial hypertension. The diagnosis of MB was retained after anatomopathological examination. The patient and his sister, who is a doctor, reported having received several sessions of radiotherapy and chemotherapy. We did not have details of the doses or the number and duration of treatments, and the files were destroyed at the Radiotherapy Center according to the French law on the management of medical archives. Physical examination revealed tattoo marks on the scalp and back for craniospinal radiotherapy. The patient has evolved well postoperatively, and he has since lived asymptomatic.

The patient presented 4 months before the admission with a frontal headache, pulsatile, which increased in frequency and intensity over time, and visual disorders marked by a permanent bilateral visual loss and double vision. He also presented with an imbalance and slurring of speech with a discreet onset and very progressive evolution. The symptoms were worsened by the appearance of hiccups and vomiting outside of meals relieving headaches. He had no seizures or other deficits. During the course of his disease, he exhibited moderate weight loss and apyrexia.

Physical examination found a conscious patient, hemodynamically stable, and his performance status was evaluated at 1. The neurological examination found a cerebellar syndrome: cerebellar ataxia with gait ataxia and a predominance of truncal ataxia and dysarthria. A slight strabismus was also noted during the examination. The rest of the neurological examination found no other relevant symptoms. Physical examination of the skin revealed multiple typical lesions of the vitiligo. Examination of the other organ systems found no abnormalities.

**Figure 1:**
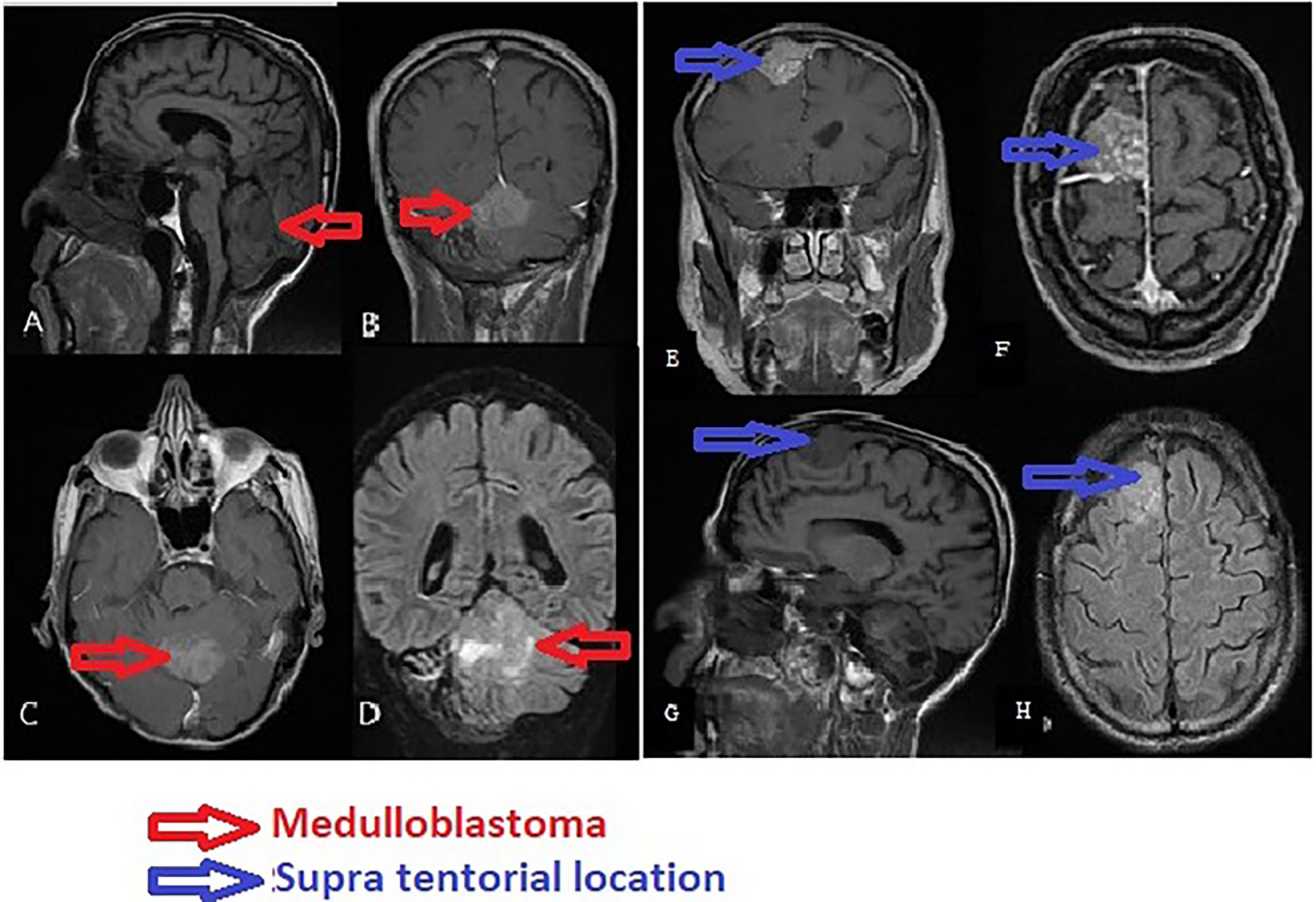
Magnetic resonance imaging of the brain showing glioblastoma of the posterior fossa and frontal meningioma. *1* Glioblastoma. **(A)** Sagittal T1. **(B)** Coronary T1-weighted post-gadolinium. **(C)** Axial T1 post-gadolinium. **(D)** Coronary fluid-attenuated inversion recovery (FLAIR). *2* Supratentorial location: probable meningioma. **(E)** Coronary T1 post-gadolinium. **(F)** Axial T1-weighted post-gadolinium. **(G)** Sagittal T1 plane showing the scalloping. **(H)** Axial FLAIR.

The initial cerebral CT scan and a cerebral MRI revealed the double location of the tumors. In the posterior fossa, the tumor was in the cerebellar vermis and the right cerebellar hemisphere, grossly limited and irregularly well enhanced after injection. The second is an extra-axial tumor, a dural-based mass, at the right paramedian level in contact with the superior sagittal sinus, which was enhanced vividly on both MRI and CT with some calcifications and scalloping appearance on the cranial vault (**Figure 1**).

According to the imaging features of the lesion of the posterior cerebral fossa and the location on the same old operating bed next to the old craniotomy, the most likely diagnosis was a recurrence of MB. Following this reasoning, we completed the examination with an MRI of the spine to rule out metastasis along the cerebrospinal fluid (CSF) outflow pathways. Medullary MRI did not reveal abnormalities. The second supratentorial lesion was probably a radiation-induced parasagittal sinus meningioma, based on suggestive imaging features of a patient undergoing radiotherapy for a long time.

We decided to operate on the lesion of the posterior fossa because it showed worse prognosis than did the supratentorial lesion. It was also the symptomatic lesion. The same surgical median suboccipital approach was used in the prone position by incising on the occipito-cervical scar. Intraoperatively, the tumor was grayish, friable, and very hemorrhagic. The follow-up was simple. The patient preserved some gait ataxia.

**Figure 2:**
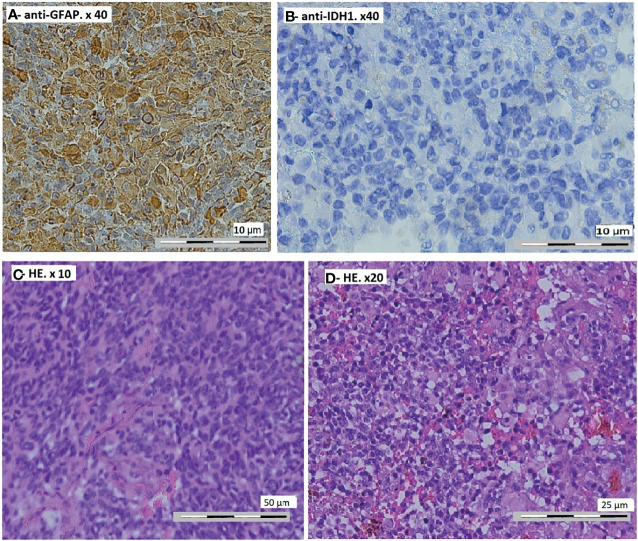
Pathological findings on the tumor. **(A)** Focal positivity of the tumor cells to anti-GFAP (glial fibrillary acidic protein). Immunohistochemistry, anti-GFAP, ×40 magnification. **(B)** Negativity of tumor cells to anti-IDH1. Anti-IDH1 immunohistochemistry (R132H), ×40 magnification. **(C)** Global value of the proliferation. Hematoxylin–eosin stain, ×10 magnification. **(D)** Diffuse tumor proliferation, poor differentiation, and rich vascularization. Hematoxylin–eosin stain, ×20 magnification.

**Figure 3:**
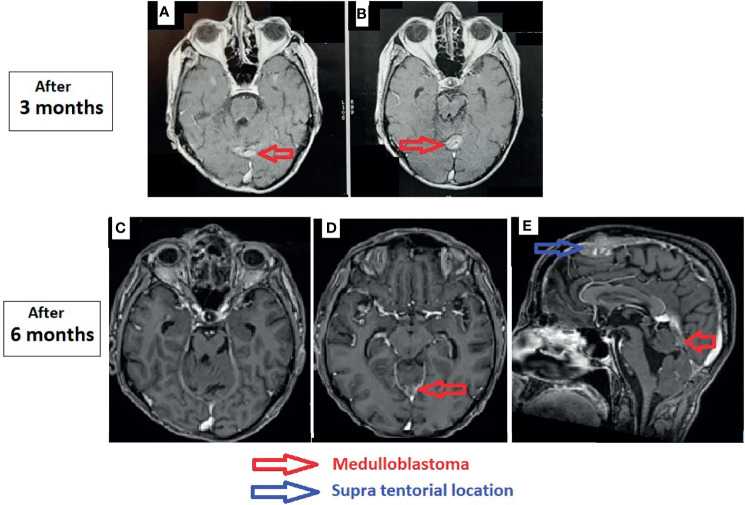
Control magnetic resonance imaging of the brain. **(A, B)** After 3 months: axial T1-weighted post-gadolinium. **(C–E)** After 6 months: axial T1-weighted post-gadolinium **(C, D)** and sagittal T1-weighted post-gadolinium **(E)**.

The anatomopathological study showed a diffuse tumor proliferation, poor differentiation, and rich vascularization. Immunohistochemistry showed a focal positivity to anti-GFAP (glial fibrillary acidic protein) and negativity of tumor cells to anti-IDH1, concluding in glioblastoma with rhabdoid characteristics (**Figure 2**). Control CT scan and MRI revealed a resection exceeding 50% of the tumor (**Figures 3A, B**). The surgery outcome was good.

During the presentation of this case at the neuro-oncology multidisciplinary consultation meeting, following the testimonies of the patient and his sister, who is a doctor, identification of the tattoo marks on the scalp and back, and recognition of the practice of the Lyon Radiotherapy Center at that time, our radiotherapists adopted the theory that the patient received a maximum dose of 54 Gy and craniospinal radiotherapy for the treatment of his MB. The prognosis of the glioblastoma was very bad. We discussed the re-irradiation and the chemotherapy. We decided to begin with the chemotherapy based on temozolomide. We then reevaluated the results and discussed the re-irradiation.

The patient received temozolomide 5 days/week, and we rediscussed the case at the meeting after three cycles and reevaluation by MRI. The residual tumor was stable, so we decided to continue with temozolomide for another three cycles and to reevaluate by MRI. We found a clear reduction of the size of the residual tumor (**Figures 3C–E**). The size of the supratentorial tumor was stable during the follow-up, with partial thrombosis of the superior longitudinal sinus at the last MRI.

The follow-up was done monthly with physical examination and a cerebral MRI every 3 months. Chemotherapy tolerance was marked by some nausea, anorexia, and grade 2 thrombocytopenia three times, temporarily interrupting resumption of the chemotherapy precisely after the second, fourth, and sixth cycles.

The patient died at home after approximately 11 months of survival. It was a sudden death, the cause of which remains unknown.

## Discussion

Glioblastoma as a long-term effect of radiotherapy is still rare in the literature. The estimated cumulative risk of radiation-induced glioblastoma (RIG) is 1.7%–2.7% at 15 years after radiation therapy. Glioblastoma is relatively more frequent following some treated tumors, such as acute lymphoblastic leukemia/lymphoma: 81% within 10 years after radiotherapy, 59% following other tumors, and 18% with benign conditions (17).

A review of the literature on RIG following MB at the same site in the posterior fossa revealed six cases (**Table 1**), since the first case reported by Kleriga et al. in 1972, and two cases of RIG following MB at the supratentorial location.

**Table 1:** Comparison of the clinical cases reported in the literature.

	Glioblastoma at the same site							Supratentorial glioblastoma	
Authors, year	Schmidbauer et al., 1987 (5)	Yang et al., 2005 (7)	Gessi et al., 2008 (8)	Hamasaki et al., 2010 (9)	Madden et al. (series), 2010 (18)	Martin et al., 2012 (10)	Our case, 2021	Pearl et al., 1980 (11)	Wang et al., 2018 (12)
Onset age (years), gender	13, M	5, M	7, M	5, M	5, M	9, M	27, M	5, M	4, M
Surgery	Total removal	Gross total resection	Total removal	Gross total resection	Gross total resection	Gross total resection	No data	Subtotal rasection	Total resection
Radiation dose (Gy), whole brain	60	36	20.8	40	23.4	23.4	Yes (no data)	30	30.6
Radiation dose (Gy), posterior fossa	–	20	59.8	–	55.8	55.8	No data	10	54
Radiation dose, spinal cord (Gy)	–	24	–	30.5	23.4	23.4	Yes (no data)	20	30.6
Chemotherapy	Vincristine and CCNU	Eight-in-one regimen (solumedrol, vincristine, lomustine, procarbazine, hydroxyurea, cisplatin, cytoxan, and cytosine arabinoside)	Methotrexate, etoposide, cyclophosphamide, carboplatinum	No chemotherapy	Pre-RT: vincristine. Post-RT: lomustine, cisplatin, and vincristine Or cyclophosphamide, cisplatin, and vincristine	CCNU (lomustine), cisplatin and vincristine	Yes (no data)	No data	No
Latency of recurrence (years)	6	10	8	35	6	5	29	13	8
Surgery	Radical resection	Subtotal removal	Partial removal	Partial removal	Tumor resection	No surgery	Subtotal resection	Left occipital lobectomy	Yes, resection
Histology of biopsy	GBM	Glioblastoma	Glioblastoma	GBM	GBM	Postmortem biopsy: GBM	GBM with rhabdoid characteristics	GBM	GBM with rhabdoid characteristics
Radiation for GBM (RT)	55 Gy at the PF	No	No	50 Gy	No	No	No	30 Gy to whole brain, 15 boosts on the PF and 30 for the spinal cord	No
Chemotherapy for GBM	Yes (no data)	Tamoxifen, irinotecan, cisplatin, and 13-*cis*-retinoic acid	Temozolomide	ACNU, procarbazine, and vincristine	No chemotherapy	Yes (no data)	Temozolomide	CCNU, procarbazine, and vincristine	Nimotuzumab, bevacizumab, and irinotecan
Follow-up	2 years, good evolution	1 year	No data	1 year	5 months, deterioration	4 months	11 months, good evolution	2 years, progressive worsening	1 y, progressive worsening
Outcome	Death, 2 years	Death, 1 year	No data	Death, 1 year	Death, 5 months	Death, 4 months	Death, 11 months	Death, 2 years	Death, 1 year

GBM, glioblastoma multiforme; ACNU, 1-(4-amino-2-methyl-5-pyrimidinyl)-methyl-3-(2-cholroethyl)-3-nitrosourea hydrochloride; PF, posterior fossa; RT, radiotherapy.

The criteria for RIG established by Cahan et al. (19) were as follows: a) the phenotypes of the primary and secondary tumors were distinctly different; b) the site of the secondary tumor was within the field of irradiation; c) the secondary tumor should be histologically diagnosed; and d) there should be a prolonged latency period between the treatment of the primary tumor and the appearance of the secondary tumor.

The biological and genetic mechanisms leading to RIG are still misunderstood (20). As well as the exact origin of these tumors, RIGs can arise from reactive astrocytes within the radiation field or from radiation-induced transformation of the original tumor cells to a different phenotypic tumor type (11, 21). This uncertainty about the origin of development was fueled by the fact that these RIGs develop in the same site as that of the original tumor (7, 11).

In 2010, Hamasaki et al. (9), based on the study by Paulino et al. (17), showed that RIG was not correlated with sex, age at radiotherapy (RT), the initial condition treated with RT, the RT dose or volume, surgery, or chemotherapy (9). Recently, Wang et al. (12) have observed that the age of patients who developed RIG was frequently younger compared with that in spontaneous high-grade gliomas. The authors concluded that patient age at first radiotherapy may be an important factor in the development of radiation-induced brain tumors. Another factor affecting the development of RIG is irradiation volume. The incidence of RIG increases with increasing irradiation volume.

Secondary tumors present a strong immunoreactivity for both GFAP and p53 (7, 22, 23). The strong immunoreactivity for p53 has been frequently associated with mutations in the *p53* gene, even if these were not detected. These mutations have been identified in radiation-induced tumors by some authors (8, 24). The secondary epithelioid/RGBM, in addition to the strong immunoreactivity for both GFAP and p53, expresses vimentin, synaptophysin, and epithelial membrane antigen (EMA) (23, 25, 26). Focal loss of the INI-1 protein has also been observed in rhabdoid tumor cells (23, 27). RGBM has poor prognosis, with a median survival from the time of diagnosis of only ∼4.9 months (23, 28).

We operated on our patient by subtotal surgical excision exceeding 50%. We added adjuvent chemotherapy based on temozolomide. After 6 months, we achieved an almost total response (near-complete disappearance of the tumor on imaging). The complications of chemotherapy were well supported with medical management. We did not use radiotherapy for our patient. The prognosis was well explained to the patient and his family, and he was aware of the very short survival described in the literature. The therapeutic plan was discussed with the patient, and he showed very good treatment compliance. Our patient died after 11 months. The cause of death is still unknown, and probably is not in relation to the glioblastoma.

A review of the literature (**Table 1**) did not reveal a management consensus for radiation-induced glioblastoma, especially after a previously irradiated MB. The number of cases reported is still very limited. This literature review allowed us to compare our therapeutic methods and results, but we remain far from validating our findings. Surgical resection is the cornerstone of treatment, and it must be as complete as possible, followed by chemotherapy in all cases. Chemotherapy is administered alone, without radiotherapy in most cases. The protocols for chemotherapy are not well defined and differ from one case to another. Radiotherapy associated with chemotherapy was used in only three cases reported in our review. Patient survival is still between a maximum of 2 years and a minimum of 4 months, with an average of 13.4 months.

Undoubtedly, radiation injury is a factor in the development of meningiomas (15), and it is the most common brain neoplasm known to be caused by ionizing radiation (14, 15). In 1953, Mann et al. published the first case of radiation-induced meningioma (RIM) (29). The average latency period between irradiation of the primary tumor and RIM is 19.5 years (range, 3.5–63 years) (30, 31). Female predominance is less marked compared to spontaneous meningioma. It is estimated at 1.29/1 in the literature (32–35). In the literature, the dose limit, above which there is a risk of developing meningioma, is controversial. Banerjee et al. (33) estimated this dose limit at 21 Gy. Nigliea et al. (36) reported a dose limit of 30 Gy.

RIM is very different from spontaneous meningioma (SM). It occurs in younger populations: the mean age at presentation has been reported as 29.2–37.9 years in patients exposed to high-dose radiation and as 45–58 years in those who received low-dose treatment, whereas SM generally arises in the fifth or sixth decade of life (32). It is also relatively more frequently multifocal (15) and more aggressive. The most common histological subtypes are meningotheliomatous, transitional, and fibroblastic. They are characterized by six distinct histological features: a high degree of cellularity, cellular pleomorphism, numerous bizarre cells, necrotic changes, increased mitotic figures, and nuclei with pseudoinclusions (32). The reported rates of recurrence vary between 18.7% and 25.6% compared to 3%–11.4% among SMs (15).

Surgical management is based on an aggressive resection of the dura mater, with a large resection margin. This is completed by removal of the bone if there is suspicion of osseous invasion with acrylic replacement (15).

## Lessons

This clinical case illustrated the need for the prolonged monitoring of patients who received treatment with radiotherapy for brain tumors, thus allowing early and adequate treatment of any abnormalities discovered during monitoring.

In the literature, the main therapeutic methods used are principally surgical resection followed by chemotherapy only without radiotherapy, or re-irradiation combined with chemotherapy.

Temozolomide retains its place in chemotherapy for radiation-induced glioblastoma.

## Data Availability Statement

The original contributions presented in the study are included in the article/supplementary material. Further inquiries can be directed to the corresponding author.

## Ethics Statement

Ethical review and approval were not required for the study on human participants in accordance with the local legislation and institutional requirements. The patients/participants provided written informed consent to participate in this study. Written informed consent was obtained from the individual(s) for the publication of any potentially identifiable images or data included in this article.

## Author Contributions

TM and AL conceived and designed the study. TM drafted the manuscript. AL critically revised the manuscript for content. All authors contributed to the article and approved the submitted version.

## Conflict of Interest

The authors declare that the research was conducted in the absence of any commercial or financial relationships that could be construed as a potential conflict of interest.

## Publisher’s Note

All claims expressed in this article are solely those of the authors and do not necessarily represent those of their affiliated organizations, or those of the publisher, the editors and the reviewers. Any product that may be evaluated in this article, or claim that may be made by its manufacturer, is not guaranteed or endorsed by the publisher.
